# Self‐Reported Sleep Duration, Napping, and Incident Heart Failure: Prospective Associations in the British Regional Heart Study

**DOI:** 10.1111/jgs.14255

**Published:** 2016-06-28

**Authors:** S. Goya Wannamethee, Olia Papacosta, Lucy Lennon, Peter H. Whincup

**Affiliations:** ^1^ Department of Primary Care and Population HealthUniversity CollegeLondonUnited Kingdom; ^2^ Population Health Research InstituteSt George's, University of LondonLondonUnited Kingdom

**Keywords:** sleep duration, daytime sleep, heart failure

## Abstract

**Objectives:**

To examine the associations between self‐reported nighttime sleep duration and daytime sleep and incident heart failure (HF) in men with and without preexisting cardiovascular disease (CVD).

**Design:**

Population‐based prospective study.

**Setting:**

General practices in 24 British towns.

**Participants:**

Men aged 60–79 without prevalent HF followed for 9 years (N = 3,723).

**Measurements:**

Information on incident HF cases was obtained from primary care records. Assessment of sleep was based on self‐reported sleep duration at night and daytime napping.

**Results:**

Self‐reported short nighttime sleep duration and daytime sleep of longer than 1 hour were associated with preexisting CVD, breathlessness, depression, poor health, physical inactivity, and manual social class. In all men, self‐reported daytime sleep of longer than 1 hour duration was associated with significantly greater risk of HF after adjustment for potential confounders (adjusted hazard ratio (aHR) = 1.69, 95% CI = 1.06–2.71) than in those who reported no daytime napping. Self‐reported nighttime sleep duration was not associated with HF risk except in men with preexisting CVD (<6 hours: aHR = 2.91, 95% CI = 1.31–6.45; 6 hours: aHR = 1.89, 95% CI = 0.89–4.03; 8 hours: aHR = 1.29, 95% CI = 0.61–2.71; ≥9 hours: aHR = 1.80, 905% CI = 0.71–4.61 vs nighttime sleep of 7 hours). Snoring was not associated with HF risk.

**Conclusion:**

Self‐reported daytime napping of longer than 1 hour is associated with greater risk of HF in older men. Self‐reported short sleep (<6 hours) in men with CVD is associated with particularly high risk of developing HF.

Numerous prospective studies and meta‐analyses of observational studies have implicated sleep duration in the development of cardiovascular disease (CVD); short or long sleep duration or both have been associated with greater risk of CVD.^1^, ^2^, ^3^, ^4^ Complaints of sleep difficulty are common in elderly adults.^5^ Daytime sleepiness that leads to napping is common in elderly adults, reflecting changes in sleep efficiency, sleep quality, and circadian sleep–wake cycles.^6^ Daytime sleepiness or excessive daytime napping has been associated with CVD in middle‐aged and older populations.^7^, ^8^, ^9^, ^10^, ^11^, ^12^ Sleep disruptions are common in individuals with heart failure (HF), which may result in excessive sleepiness during the daytime,^13^ but prospective studies on the relationship between sleep duration and daytime napping and sleepiness and incident HF in people without HF are limited. Although three studies have shown daytime sleepiness or insomnia symptoms to be associated with greater HF risk,^7^, ^14^, ^15^ two other studies showed no association between sleep duration and HF risk.^3^, ^16^ Although previous research has suggested that impaired sleep may also affect prognosis after CVD,^17^, ^18^ the association between sleep duration and daytime sleep with risk of HF specifically in those with a history of CVD has been little studied. The current study examined the association between self‐reported sleep duration, daytime sleep, and risk of incident HF in a cohort study of older British men with and without preexisting CVD.

## Subjects and Methods

The British Regional Heart Study is a prospective study of CVD involving 7,735 men aged 40–59 selected from the age and sex registers of one general practice in each of 24 British towns and screened between 1978 and 1980.^19^ In 1998–2000, all surviving men (N = 5,447), now aged 60–79, were invited for a 20‐year follow‐up examination; 4,252 (77% of survivors) attended. All relevant local research ethics committees provided ethical approval. All men provided informed written consent to the investigation, which was conducted in accordance with the Declaration of Helsinki. In 2003, a postal questionnaire was sent to all survivors, then aged 63–82, that asked for information on medical history, lifestyle changes, and sleep habits; 3,982 men responded (81% response rate). Of these, 259 with a history of a doctor diagnosis of HF were excluded, leaving 3,723 for analysis.

### Cardiovascular Risk Factors and Morbidity

In the 2003 questionnaire, detailed questions were asked on smoking habits, physical activity, and body weight.^20^ Participants were classified into manual and nonmanual workers based on the longest‐held occupation of subjects at study entry using the Registrar General Social Class Classification. Body mass index (BMI) was calculated as body weight divided by height squared (kg/m^2^). Height was extrapolated from height measured during the physical examination in 1998–2000. Subjects were asked to report a doctor diagnosis of heart attack, stroke, heart failure, depression, high blood pressure, or high blood cholesterol and to report symptoms of breathlessness and treatment to lower blood pressure. They were also asked to describe their health status as excellent, good, fair, or poor.

### Self‐Reported Sleep

In the 2003 questionnaire, self‐reported nighttime sleep duration and daytime sleep were determined based on answers to the questions: “On average how many hours sleep do you have each night?” and “On average how much sleep do you have during the daytime?” The men responded with duration of sleep in hours and minutes. They were also asked whether they snored (yes regularly, yes occasionally, no, don't know).

### Follow‐Up

All men have been followed from initial examination (1978–1980) for cardiovascular morbidity 21 and all‐cause mortality, with near‐complete follow‐up (99%) of the cohort for morbidity and mortality. In the present analyses, all‐cause mortality and morbidity events are based on 9 years of follow‐up from 2003–2012. Mortality data was collected through the established “tagging” procedures that the National Health Service registers provide. Nonfatal myocardial infarction (MI) was diagnosed according to World Health Organization criteria. Evidence of nonfatal MI and HF was obtained according to ad hoc reports from general practitioners supplemented by biennial reviews of practice records (including hospital and clinic correspondence) to the end of the study period. Incident nonfatal HF was based on a confirmed doctor diagnosis of HF from primary care records and where possible verified using details of available clinical information from primary and secondary care records (including symptoms, signs, investigations, and treatment response) to ensure that the diagnosis was consistent with current recommendations on HF diagnosis.^22^ Incident HF included incident nonfatal HF and death from HF as the underlying cause (*International Classification of Diseases, Ninth Revision*, code 428).

### Statistical Methods

Nighttime sleep duration was categorized into five groups (<6, 6, 7, 8, ≥9 hours; 7 hours was used as the reference group. Daytime napping was categorized into four groups (none, <1, 1, >1 hour). Cox proportional hazards models were used to assess hazard ratios (HRs) (relative risk), adjusting for factors known to be associated with HF risk, including smoking (never, ex, current), social class (manual, nonmanual), physical inactivity (yes, no), previous MI (yes, no), stroke (yes, no), diabetes mellitus (yes, no), use of antihypertensive treatment (yes, no), breathlessness (yes, no), BMI, and poor health (yes, no). To assess whether the association between duration of sleep and daytime sleep and incident HF may be due to the development of incident MI, incident CHD was adjusted for by fitting CHD as a time‐dependent covariate. Subsidiary analyses were performed stratified according to preexisting doctor‐diagnosed CVD (MI, stroke). All analyses were performed using SAS version 9.3 (SAS Institute, Inc., Cary, NC).

## Results

During the follow‐up of 9 years, there were 199 incident HF cases (rate 7.3/1,000 person‐years) in the 3,723 men with no diagnosed HF. Mean±standard deviation reported nighttime sleep in the study was 7.0 ± 1.3 hours.

### Baseline Characteristics According to Self‐Reported Sleep Variables

Men who reported 7–8 hours of sleep generally had the lowest prevalence of adverse risk factors. Men who reported short sleep duration (<6 hours) had by far the highest prevalence of poor health, preexisting CVD, breathlessness, and history of depression (Table 1). Those who reported long sleep duration (≥9 hours) also had high rates of preexisting CVD. Men who reported daytime napping of longer than 1 hour had the highest prevalence of adverse risk factors and ill health. These men were also more likely to report short sleep duration at night and regular snoring.

**Table 1:** Baseline Characteristics According to Self‐Reported Nighttime Sleep Duration and Daytime Napping in 3,723 Men without Heart Failure

Characteristic	Self‐Reported Nighttime Sleep Duration, Hours						Self‐Reported Daytime Napping, Hours				
	<6, n = 348	6, n = 767	7, n = 1,064	8, n = 1,188	≥9, n = 281	*P*‐Value	0, n = 1,889	<1, n = 679	1, n = 869	>1, n = 286	*P*‐Value
Age, mean ± SD	68.5 ± 5.5	68.3 ± 5.7	67.5 ± 5.2	68.1 ± 5.3	69.5 ± 5.5	<.001	67.4 ± 5.2	68.4 ± 5.2	69.1 ± 5.3	69.4 ± 6.1	<.001
Obese, n (%)	45 (12.9)	83 (10.8)	103 (9.7)	129 (10.9)	26 (9.3)	.46	189 (10.0)	61 (9.0)	103 (11.9)	41 (14.3)	.04
Smoker, n (%)	33 (9.5)	76 (9.9)	93 (8.7)	108 (9.1)	33 (11.7)	.61	166 (8.8)	35 (5.2)	106 (12.2)	45 (15.7)	<.001
Physically inactive, n (%)	167 (48.0)	280 (36.5)	341 (32.1)	360 (30.3)	110 (39.1)	<.001	577 (30.6)	180 (26.5)	368 (42.4)	152 (53.2)	<.001
Manual worker, n (%)	214 (61.5)	414 (54.0)	488 (45.9)	611 (51.4)	156 (55.5)	<.001	1,054 (55.8)	271 (39.9)	436 (50.2)	176 (61.5)	<.001
Heavy drinker, n (%)	11 (3.2)	20 (2.6)	26 (2.4)	26 (2.2)	11 (3.9)	.51	48 (2.5)	9 (1.3)	28 (3.2)	10 (3.5)	.08
Poor health, n (%)	40 (11.5)	26 (3.4)	20 (1.9)	23 (1.9)	7 (2.5)	<.001	42 (2.2)	6 (0.9)	36 (4.1)	35 (12.2)	<.001
Hypertension, n (%)	147 (42.2)	290 (37.8)	375 (35.2)	461 (38.8)	117 (41.6)	.09	692 (36.6)	248 (36.5)	358 (41.2)	116 (40.6)	.08
High cholesterol, n (%)	72 (20.7)	170 (22.2)	216 (20.3)	283 (23.8)	55 (19.6)	.25	392 (20.8)	174 (25.6)	180 (20.7)	64 (22.4)	.05
Treated hypertension, n (%)	131 (37.6)	263 (34.3)	337 (31.7)	440 (37.0)	103 (36.7)	.06	615 (32.6)	229 (33.7)	335 (38.6)	109 (38.1)	.01
Diabetes mellitus, n (%)	35 (10.1)	73 (9.5)	95 (8.9)	108 (9.1)	33 (11.7)	.66	157 (8.3)	67 (9.9)	89 (10.2)	40 (14.0)	.01
Preexisting cardiovascular disease, n (%)	83 (23.9)	130 (17.0)	176 (16.5)	188 (15.8)	62 (22.1)	.002	297 (15.7)	114 (16.8)	177 (20.4)	69 (24.1)	<.001
Breathlessness, n (%)	106 (30.5)	126 (16.4)	103 (9.7)	147 (12.4)	44 (15.7)	<.001	213 (11.3)	75 (11.1)	168 (19.3)	82 (28.7)	<.001
History of depression, n (%)	41 (13.9)	70 (10.1)	67 (6.9)	81 (7.5)	27 (10.8)	<.001	132 (7.8)	57 (9.0)	66 (8.4)	39 (15.9)	<.001
Snore regularly, n (%)	87 (25.7)	144 (19.0)	210 (19.9)	228 (19.5)	39 (14.1)	.01	323 (17.8)	134 (19.9)	187 (21.7)	73 (26.1)	<.001
Nap at least 1 hr/day, n (%)	153 (44.0)	276 (36.0)	293 (27.5)	331 (27.9)	95 (33.8)	<.001					
Average number of night time sleep, mean ± SD							7.1 ± 1.2	7.1 ± 1.1	7.0 ± 1.3	6.7 ± 1.7	<.001
<6 hours sleep, n (%)							147 (7.8)	48 (7.1)	97 (11.2)	56 (19.6)	<.001

Data on nighttime sleep duration were not available for 75 men.

SD = standard deviation.

### Self‐Reported Sleep Duration, Daytime Sleep, and Incident HF

Only self‐reported short nighttime sleep was associated with greater risk of HF, although this was largely due to adverse characteristics associated with short sleep because adjustment for age, social class, BMI, smoking, diabetes mellitus, physical activity, treated hypertension, breathlessness, preexisting CVD, and poor health attenuated the association (Table 2). Self‐reported daytime napping of longer than 1 hour was associated with significantly greater risk of HF that remained even after adjustment for the above potential confounders. When stratified according to preexisting CVD status, no association was found between sleep duration and HF risk in those with no history of CVD (Table 3), although in men with preexisting CVD, a significant U‐shaped association was observed, with short and long sleep associated with greater HF risk even after adjustment for potential confounders (quadratic trend *P* = .03). Daytime napping of longer than 1 hour was associated with greater risk of HF particularly in men without CVD. This persisted even after taking into account incident CHD. Self‐reported regular snoring was not associated with risk of HF in either group.

**Table 2:** Self‐Reported Nighttime Sleep Duration, Daytime Napping, Snoring, and Risk of Incident Heart Failure in Older Men with No History of Doctor‐Diagnosed Heart Failure

Factor	n	Rate/1,000 Person‐Years (n)	Hazard Ratio (95% Confidence Interval)	
			Age Adjusted	Adjusted^a^
Self‐reported nighttime sleep duration, hours				
<6	348	9.7 (25)	1.53 (0.95–2.45)	1.26 (0.77–2.05)
6	767	7.4 (45)	1.16 (0.78–1.72)	1.09 (0.73–1.63)
7	1,064	6.1 (53)	1.00	1.00
8	1,188	5.8 (55)	0.92 (0.63–1.34)	0.89 (0.61–1.29)
≥9	281	8.1 (17)	1.19 (0.69–2.06)	1.07 (0.62–1.86)
Self‐reported daytime napping, hours				
0	1,889	5.7 (88)	1.00	1.00
<1	679	5.9 (33)	0.94 (0.63–1.41)	0.95 (0.63–1.42)
1	869	8.3 (55)	1.30 (0.93–1.83)	1.18 (0.84–1.66)
>1	286	12.4 (23)	1.99 (1.25–3.15)	1.69 (1.06–2.71)
Self‐reported snoring				
No	462	8.0 (28)	1.00	1.00
Occasionally	1,868	5.8 (88)	0.75 (0.55–1.02)	0.76 (0.55–1.04)
Regularly	717	6.3 (37)	0.89 (0.60–1.33)	0.82 (0.55–1.23)

Data on nighttime sleep duration were not available in 75 men. Data on snoring were not available in 90 men, who were excluded from the analysis on snoring.

a Adjusted for age, type of work, body mass index, smoking, diabetes mellitus, physical activity, treated hypertension, breathlessness, preexisting myocardial infarction, stroke, poor health.

**Table 3:** Self‐Reported Nighttime Sleep Duration, Daytime Napping, and Risk of Incident Heart Failure in Older Men with and without Preexisting Cardiovascular Disease (CVD; Myocardial Infarction or Stroke)

Factor	Rate/1,000 Person‐Years (n)	Hazard Ratio (95% Confidence Interval)	
		Age Adjusted	Adjusted^a^
No preexisting CVD (n = 3,066)			
Self‐reported nighttime sleep duration, hours			
<6 (n = 265)	5.9 (12)	1.00 (0.53–1.91)	0.77 (0.40–1.51)
6 (n = 637)	5.6 (29)	0.97 (0.60–1.56)	0.86 (0.53–1.40)
7 (n = 888)	5.5 (41)	1.00	1.00
8 (n = 1,000)	4.7 (38)	0.82 (0.53–1.27)	0.77 (0.49–1.19)
≥9 (n = 219)	5.9 (10)	0.94 (0.47–1.88)	0.85 (0.42–1.70)
Self‐reported daytime napping, hours			
0 (n = 1592)	4.5 (59)	1.00	1.00
<1 (n = 565)	3.8 (18)	0.79 (0.47–1.34)	0.79 (0.45–1.35)
1 (n = 692)	7.2 (39)	1.46 (0.97–2.20)	1.31 (0.87–1.98)
>1 (n = 217)	10.8 (16)	2.22 (1.28–3.86)	1.79 (1.01–3.17)
Preexisting CVD (n = 657)			
Self‐reported nighttime sleep duration, hours			
<6 (n = 83)	23.9 (13)	2.76 (1.26–6.06)	2.91 (1.31–6.45)
6 (n = 130)	17.2 (16)	1.82 (0.86–3.85)	1.89 (0.89–4.03)
7 (n = 176)	9.4 (12)	1.00	1.00
8 (n = 188)	12.6 (17)	1.29 (0.63–2.71)	1.29 (0.61–2.71)
≥9 (n = 62)	17.7 (7)	1.87 (0.74–4.76)	1.80 (0.71–4.61)
Self‐reported daytime napping, hours			
0 (n = 297)	13.5 (29)	1.00	1.00
<1 (n = 114)	17.3 (15)	1.16 (0.62–2.17)	1.16 (0.61–2.20)
1 (n = 177)	13.3 (16)	0.90 (0.49–1.66)	0.88 (0.47–1.64)
>1 (n = 69)	19.0 (7)	1.40 (0.61–3.20)	1.35 (0.58–3.10)

Data on nighttime sleep duration were not available in 75 men.

a Adjusted for age, social class, body mass index, smoking, physical activity, diabetes mellitus, treated hypertension, breathlessness, and poor health.

The association between self‐reported daytime napping and HF risk was largely seen in obese men (BMI ≥30.0 kg/m^2^) (adjusted HR (aHR) = 4.27, 95 CI = 1.58–11.59). No association was found in nonobese men (aHR = 1.27, 95% CI = 0.73–2.22; *P* = .008).

## Discussion

In this study of older British men, only reported excessive daytime sleeping (>1 hour), which was prevalent in 7% of the men, was associated with significantly greater risk of HF than not napping; this was seen particularly in men without preexisting CVD and in obese men. Self‐reported nighttime sleep duration was associated with HF only in men with preexisting CVD. There was no association between self‐reported habitual snoring and HF, which is consistent with findings of other studies.^7^, ^18^ The findings of the current study support and extend the findings of previous studies that have implicated daytime sleepiness^7^ and sleep disturbance in HF.^14^, ^15^

### Self‐Reported Nighttime Sleep Duration and HF

No association was found between self‐reported nighttime sleep duration and HF in the overall population, which is consistent with findings from two other large studies of men and women,^3^, ^16^ although in men with CVD, a U‐shaped relationship was seen, with short sleep in particular and to a lesser extent long nighttime sleep related to greater HF risk. No association was seen in the larger group of men without CVD. Obstructive sleep apnea (OSA), which is common in individuals with CVD, is a major cause of sleep disturbance and short sleep^23^ and has been linked to incident HF.^24^ The higher risk of HF associated with short sleep in men with preexisting CVD may reflect the high rates of OSA. Short sleep duration after MI may also be a consequence of more‐severe underlying cardiac dysfunction leading to hypoxia and greater risk of HF. Long nighttime sleep duration in men with preexisting CVD may be a consequence of the coexistence of a number of comorbidities such as chronic kidney disease and stroke, which puts these men at higher risk of HF, or it may reflect depression, which has been linked to HF risk.^25^

### Self‐Reported Daytime Sleep and HF

Self‐reported daytime sleep of longer than 1 hour was associated with shorter nighttime sleep, obesity, depression, breathlessness, prevalent CVD, and overall ill health, although the association between self‐reported daytime sleep and HF was seen particularly in those with no CVD and after adjustment for these factors. These findings are consistent with those reported in the Cardiovascular Health Study, which found daytime sleepiness to be associated with HF.^7^ In contrast, in a Japanese cohort study, daytime napping (yes, no) was not associated with HF after adjustment for confounders, although duration of daytime napping was not examined.^8^

The association between daytime sleep and HF could reflect insufficient sleep or disturbed sleep,^6^ which have been associated with HF risk,^14^, ^15^ although short nighttime sleep duration, which is a characteristic of insomnia, was not associated with HF in men without CVD, in whom daytime napping was more strongly associated with HF risk. The Cardiovascular Health Study found daytime sleepiness but not actual sleep disturbance to be associated with HF.^7^ Another possible explanation for the relationship between daytime sleep and HF may be OSA, which is commonly associated with snoring and daytime sleepiness.^23^ Obesity is a major correlate of OSA, and the finding that daytime sleep was associated with HF only in obese men is consistent with this explanation.

### Strengths and Limitations

The strengths of the study include a study population socially representative of the United Kingdom and the exceptionally high follow‐up rates in the British Regional Heart Study. There are several limitations of this study. It is based on a cohort of older (60–79) men, and the results need further confirmation in women and other older and middle‐aged populations. The findings are based on self‐reported sleep patterns measured at one point only, and the possibility cannot be excluded that nighttime sleep duration and time spent daytime napping may have been misclassified, raising the possibility that the strength of the associations between sleep duration, napping, and HF have been underestimated. It is not known whether the daytime napping was voluntary, but self‐reported daytime napping of longer than 1 hour per day has been shown to be associated with markedly greater mortality.^26^ Information was not available on time spent in bed, but the average self‐reported nighttime sleep of 7 hours in this study is comparable with the findings of another UK population study of older adults that reported 6.9 hours average sleep.^27^ Nighttime sleep duration was based on average duration of nighttime sleep; no measures of nighttime sleep interruptions or information on sleep apnea and insomnia, which could affect sleep duration and influence HF risk, were available. The current findings are based on doctor‐diagnosed HF, which is likely to underestimate the true incidence of HF in this study population, although the determinants of this HF outcome in this study population (including obesity, N‐terminal of the prohormone brain natriuretic peptide, social class)^28^, ^29^, ^30^ generally accord with prior data and suggest that the HF outcome used was valid.

## Conclusion

Self‐reported daytime sleep of longer than 1 hour is associated with significantly greater risk of HF in older men. These findings point to the need for healthcare providers to discuss daytime sleep patterns with older adults. In men with CVD, self‐reported short nighttime sleep in particular is associated with greater risk of developing HF. Evaluation of sleep disturbances in older men with CVD may identify treatable conditions such as sleep apnea, cardiac dysfunction, or depression, which have potential implications for HF prevention.
